# High KIF2A expression predicts unfavorable prognosis in diffuse large B cell lymphoma

**DOI:** 10.1007/s00277-017-3047-1

**Published:** 2017-06-14

**Authors:** Yaping Zhang, Xuefen You, Hong Liu, Mengqi Xu, Qingxiu Dang, Li Yang, Jianfei Huang, Wenyu Shi

**Affiliations:** 1 Department of Hematology, Affiliated Hospital of Nantong University, Nantong, Jiangsu 226001 China; 2 Clinical biological sample library, Department of Pathology, Affiliated Hospital of Nantong University, Nantong, Jiangsu 226001 China

**Keywords:** Diffuse large B cell lymphoma, KIF2A, Prognostic marker

## Abstract

Kinesin family member 2A (KIF2A), a conserved motor protein, plays a critical role in the pathogenesis and prognosis of several malignant tumors. The aim of the present study was to investigate KIF2A expression in diffuse large B cell lymphoma (DLBCL), evaluate the association between KIF2A expression and the clinical parameters of the disease, and determine its prognostic value. KIF2A expression was evaluated in 134 DLBCL and 57 reactive hyperplasia samples using immunohistochemistry on a tissue microarray. The correlations between KIF2A expression with clinical parameters and prognosis were estimated using univariate and multivariate analyses. The expression of KIF2A was significantly higher in DLBCL tissue samples compared with those from subjects with reactive hyperplasia (*P*=0.002). Furthermore, increased expression of KIF2A protein in DLBCL was related to Ann Arbor stage (*P*=0.027) and international prognostic index (IPI) score (*P*=0.01). The survival analysis showed that KIF2A expression (*P*=0.016), serum LDH level (*P*=0.049), and IPI score (*P*<0.001) were independent prognostic markers for DLBCL. Our findings also confirmed that downregulating KIF2A expression decreased tumor cell viability, accompanied by downregulation of pAKT levels. Taken together, these data provide the first evidence that increased KIF2A expression predicts poor prognosis in patients with DLBCL, and a rationale for treatment of DLBCL by targeting KIF2A.

## Introduction

Diffuse large B cell lymphoma (DLBCL), the aggressive subtype of malignant lymphomas, accounts for 30–40% of all lymphomas. DLBCL patients have highly variable outcomes because of the genetic abnormalities and clinical features associated with the disease [1]. Encouraging advancements in the treatment of DLBCL have been made since the R-CHOP regimen (rituximab, cyclophosphamide, adriamycin, vincristine, and prednisone) was adopted in clinical practice. However, treatment remains suboptimal in roughly 30% of DLBCL patients [2]. Therefore, further studies which investigate biomarkers that can predict prognosis and promote the development of novel treatment options are required.

The kinesin-13 family, which includes kinesin family member 2A (KIF2A), KIF2B, and KIF2C/MCAK, is microtubule depolymerases that play a critical role in mitotic activity [3, 4]. These proteins regulate cytokinesis, mitotic spindle formation, and cell division [5, 6]. Among them, KIF2A localizes to centrosomes during mitotic progression and is essential for chromosome movement and bipolar spindle assembly [7]. Specific siRNA- or antibody-mediated knockdown of KIF2A expression results in the formation of monopolar spindles and arrest of cell cycle progression [8, 9]. Prior studies demonstrated that overexpression of KIF2A may be involved in the carcinogenesis of breast cancer [10], squamous cell carcinoma of the oral tongue (SCCOT) [11], colorectal cancer [12], and ovarian cancer [13]. However, to date, the relationship between KIF2A and DLBCL remains unknown. Herein, we show that the expression of KIF2A protein was elevated in DLBCL patients and was correlated with adverse clinicopathological features and poor prognosis. These results indicate that dysregulation of KIF2A is involved in the progression of DLBCL and may serve as a prognostic biomarker of the disease.

## Material and methods

### Sample collection

Between 2003 and 2010, the Department of Pathology at the Affiliated Hospital of Nantong University collected tumor tissue samples from 134 patients with DLBCL. Additionally, tissue samples collected from 57 sex- and age-matched subjects with reactive hyperplasia were used as controls. Histological type was confirmed in all patients by two independent pathologists. Histologic diagnosis of DLBCL patients was established according to the World Health Organization classification [14]. Clinical data, including patient age, sex, hemoglobin level, B symptoms, Ann Arbor stage, serum lactate dehydrogenase (LDH) level, international prognostic index (IPI) score, and 5-year follow-up period after chemotherapy were collected. Immunochemotherapy consisted of six to eight cycles of the standard R-CHOP regimen. This study was approved by the Ethics Committee of the Affiliated Hospital of Nantong University.

### Tissue microarray and immunohistochemical analysis

A total of 134 formalin-fixed and paraffin-embedded DLBCL tissue samples and 57 reactive hyperplasia tissue samples were obtained from the Affiliated Hospital of Nantong University between 2003 and 2010. We used tissue microarray analysis to test a representative 2-mm core of the tissue samples from each DLBCL patient (Quick-Ray, UT06, UNITMA, Korea) as previously described [15].

Immunohistochemistry was conducted on 4-μm-thick paraffin sections as previously described [16]. Briefly, tissue sections were deparaffinized and rehydrated with 100% xylene and an ethanol gradient. Subsequently, the sections were washed and subjected to antigen retrieval followed by blockade of endogenous peroxidase activity. Samples were then incubated with overnight at 4 °C in a humidified chamber. Phosphate-buffered saline was used as the negative control. Next, the sections were washed three times and incubated with a biotinylated anti-mouse secondary antibody, followed by treatment with 3,3-diaminobenzidine tetrahydrochloride substrate with horseradish peroxidase. Hematoxylin was used for counterstaining. The following antibodies were used in immunohistochemistry: KIF2A monoclonal primary antibody (Abcam, 5 μg/m), MYC (Abcam, cutoff 50%), BCL2 (ZSGB-BIO, cutoff 70%), CD10 (R&D Systems, cutoff 30%), BCL6 (Abcam, cutoff 50%), and MUM1 (Abcam, cutoff 50%). The cell-of-origin was classified according to the Hans algorithm. The KIF2A immunostaining score of each sample was evaluated independently by two qualified pathologists. The percentage of KIF2A-positive cells was scored (0–100%). Staining intensity was scored according to four levels: 0, 1, 2, and 3 (i.e., negative and weak to strong intensity). Both the percentage of positive cells and staining intensity contributed to the final evaluation of KIF2A expression. To determine the specific cutoff value for the KIF2A protein expression score, which was considered to be significant for the overall survival of patients, we used the X-tile software (The Rimm Lab at Yale University; http://www.tissuearray.org/rimmlab) as previously described [17]. The degree of KIF2A staining in samples from patients with DLBCL and those with reactive hyperplasia was quantified using a two-level grading system. The final staining scores of KIF2A were defined as follows: low expression, 0–90; high expression, 91–300.

### Cells

The DLBCL cell line (SU-DHL-8) was purchased from American Type Culture Collection (Bethesda, MD, USA). Cells were maintained in RPMI-1640 medium supplemented with 10% heat-inactivated fetal bovine serum, 100 U/mL penicillin, 100 μg/mL streptomycin, and 2 mM L-glutamine in a humidified incubator with 5% CO_2_ at 37 °C.

### Cell transfection and assessment of viability by MTS assay

SU-DHL-8 cells were transfected with shRNA targeting KIF2A, or a control shRNA (Cyagen Biosciences Inc. Guangzhou, China) using electroporation and an Amaxa 4D Nucleofector System according to the manufacturer’s instructions (Lonza,Walkersville, MD) [18]. After transfection, cells were seeded in 96-well plates and incubated at 37 °C for 48 h. Cell viability was assessed by MTS assay. Absorbance values were measured spectrophotometrically at 490 nm.

### Western blot

Protein was extracted from harvested cells using lysis buffer. A total of 30 μg of protein was loaded in each lane of 10% sodium dodecyl sulfate polyacrylamide gels, and Western blot was performed as previously described [19]. Antibodies against AKT (9272) and pAKT (Ser473; 4051) were purchased from the Cell Signaling Technology. β-actin was used as the loading control.

### Statistical analysis

The correlations between KIF2A expression and clinical parameters were determined using the chi-square test. Univariate and multivariate analyses were performed using Cox’s regression models. Survival was evaluated using Kaplan-Meier curves. Data were analyzed using STATA Version 12.0 (Stata Corporation, College Station, TX, USA). *P* < 0.05 was considered statistically significant.

## Results

### Clinical characteristics

Clinical and laboratory findings of the 134 patients with DLBCL are summarized in Table 1. Among the patients, there were 56 females and 78 males, with median age of 58 years (range 17–78 years). According to the Hans algorithm, 72 cases (53.73%) were diagnosed with the germinal center B cell (GCB) subtype of DLBCL, while 62 cases (46.27%) were diagnosed with the non-GCB subtype. According to immunohistochemistry, the incidence of MYC and BCL2 expression was 66.42% (89/134) and 47.37% (63/134), respectively. Moreover, 31.34% (42/134) of patients showed both MYC and BCL2 expression.

**Table 1:** Correlation of the high Kif2a expression with clinicopathologic characteristics in DLBCL

Clinicopathologic characteristics	No. of patients	High Kif2a expression no. (%) of patients	*X* ^2^	*P*
Gender				
Female	56	33 (58.93)	1.5193	0.218
Male	78	54 (69.23)		
Age				
≤60 years	69	41 (59.42)	1.8930	0.169
>60 years	65	46 (70.77)		
B symptoms				
No	96	61 (63.54)	0.2846	0.594
Yes	38	26 (68.42)		
Ann Arbor stage				
I to II	89	52 (58.43)	4.9146	0.027
III to IV	45	35 (77.78)		
Serum LDH level				
Normal or lower	72	49 (68.06)	0.6695	0.413
High than normal	62	38 (61.29)		
Hb				
≥120 g/L	80	51 (63.75)	0.1204	0.729
<120 g/L	54	36 (66.67)		
IPI score				
Low to intermediate low risk (0–2)	68	37 (54.41)	6.7014	0.010
Intermediate high to high risk (3–5)	66	50 (75.76)		

### The expression of KIF2A in DLBCL

**Fig. 1:**
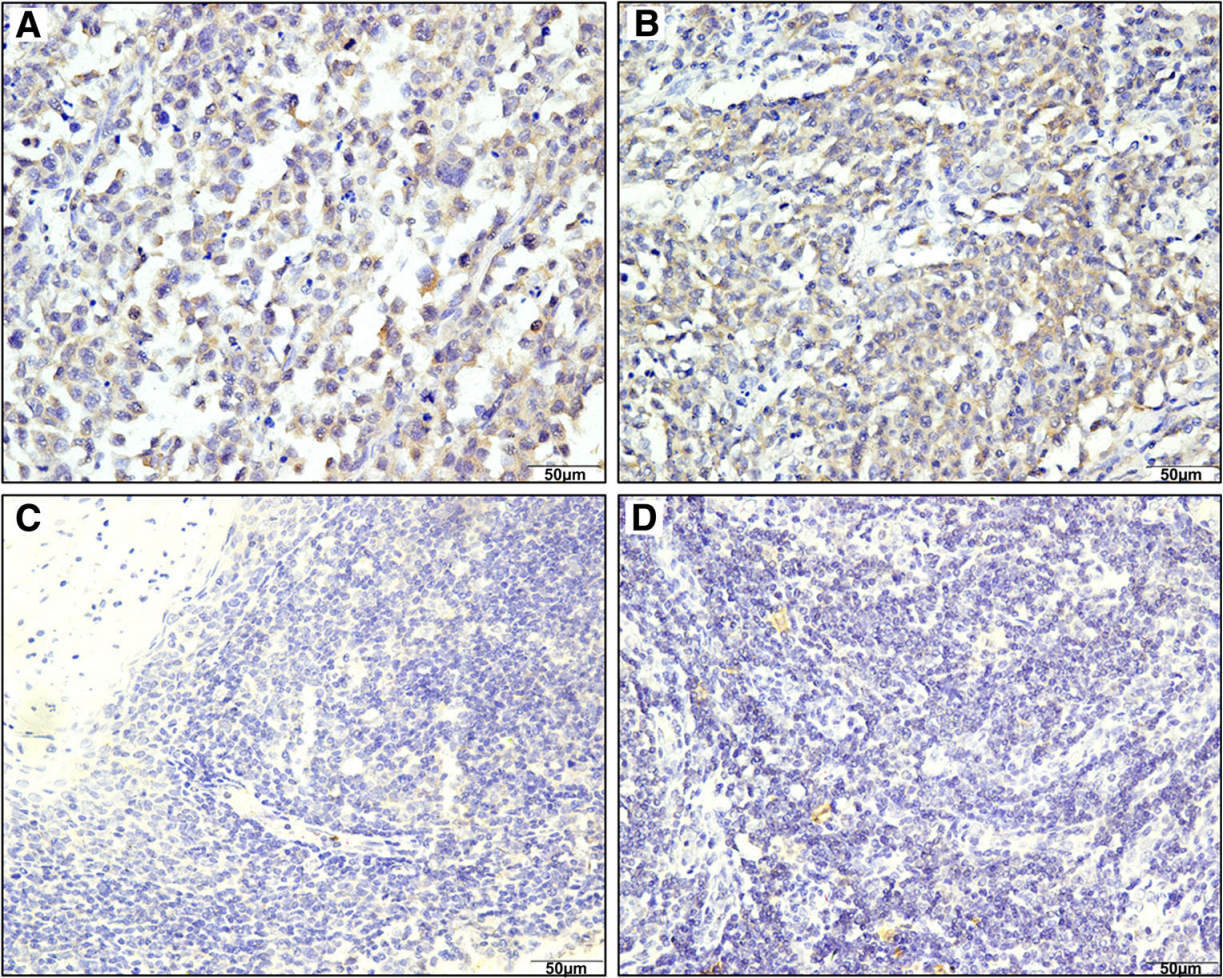
Expression of KIF2A protein in DLBCL patients and reactive hyperplasia using tissue microarray cores. Representative positive immunohistochemical staining of KIF2A in two DLBCL patients (**a** and **b**). Weakly positive staining of KIF2A in two reactive lymph nodes(**c** and **d**) (original magnification ×400)

The levels of KIF2A in DLBCL tissue samples were evaluated by immunohistochemistry. A representative image demonstrating the typical immunohistochemical staining pattern of KIF2A is shown in Fig. 1. The lymphoma tissues showed stronger staining and more positively stained cells. In total, 64.93% (87/134) of tissue samples from DLBCL patients and 40.35% (23/57) from those with reactive hyperplasia showed high expression of KIF2A (*χ* ^2^ = 9.8879, *P* = 0.002).

### The association between KIF2A expression and clinicopathological features in DLBCL

To assess the association between KIF2A expression and clinicopathological features, we analyzed 134 DLBCL patients. As shown in Table 1, an increased expression of KIF2A in DLBCL was significantly associated with Ann Arbor stage (*χ* ^2^ = 4.9146, *P* = 0.027) and IPI score (*χ* ^2^ = 6.7014, *P* = 0.01), whereas no obvious correlation was found for sex, age, B symptoms, serum LDH level, or hemoglobin level. Moreover, no similar correlations were observed between high expression of KIF2A and these clinical features in reactive hyperplasia (data not shown).

### Survival analysis of DLBCL patients

**Fig. 2:**
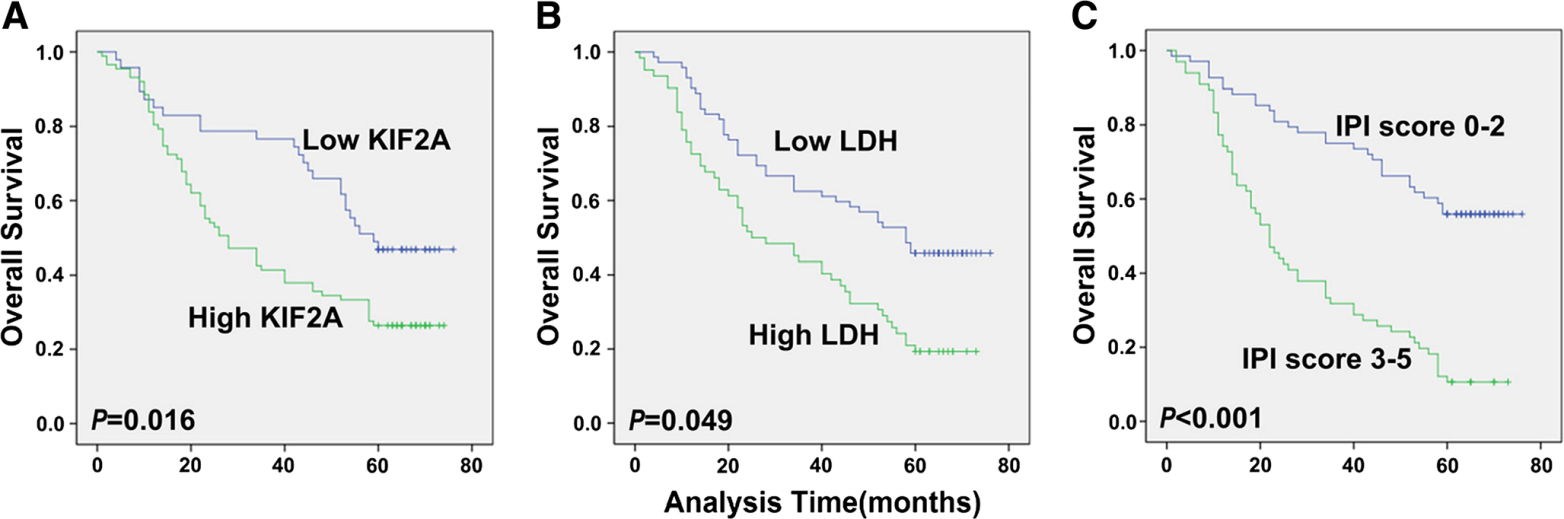
Kaplan-Meier survival curves of DLBCL patients. **a** DLBCL patients with high levels of KIF2A expression had significantly lower rate of survival than those with low KIF2A expression. **b** Patients with high LDH level showed demonstrably poorer survival than those with low LDH level. **c** Patients with low IPI score had better survival than those with high score

Univariate Cox regression analysis for all parameters showed that high KIF2A expression was significantly associated with poor survival in patients with DLBCL (*P* = 0.008) (Table 2). Serum LDH level (*P* = 0.001), Ann Arbor stage (*P* = 0.010), patient age (*P* = 0.028), and IPI score (*P* < 0.001) were implicated in the poor survival of DLBCL patients. The multivariate analysis showed that KIF2A expression (*P* = 0.016), serum LDH level (*P* = 0.049), and IPI score (*P* < 0.001) were robust prognostic markers for patients with DLBCL (Table 2). The Kaplan-Meier survival curves further demonstrated that the high KIF2A expression group exhibited remarkably shorter life expectance compared with the low KIF2A expression group. Similar results were also observed for serum LDH and IPI score (Fig. 2).

**Table 2:** Univariate and multivariate analysis of prognosis in DLBCL for 5 years overall survival

	Univariate analysis	Multivariate analysis		
	*P* > |*z*|	HR	*P* > |*z*|	95%CI
Kif2a expression (high vs low)	0.008	1.828	0.016	1.119–2.984
Gender (female vs male)	0.683			
Age (≤60 vs >60 years)	0.028			
B symptoms (no vs yes)	0.393			
Ann Arbor stage (I, II vs III, IV)	0.010			
Serum LDH level (normal or low vs high)	0.001	1.564	0.049	1.003–2.441
Hb (≥120 vs <120 g/L)	0.481			
IPI score (0–2 vs 3–5)	<0.001	3.114	<0.001	1.950–4.972

### Downregulation of KIF2A inhibits lymphoma cell proliferation and is related to the AKT signaling pathway

**Fig. 3:**
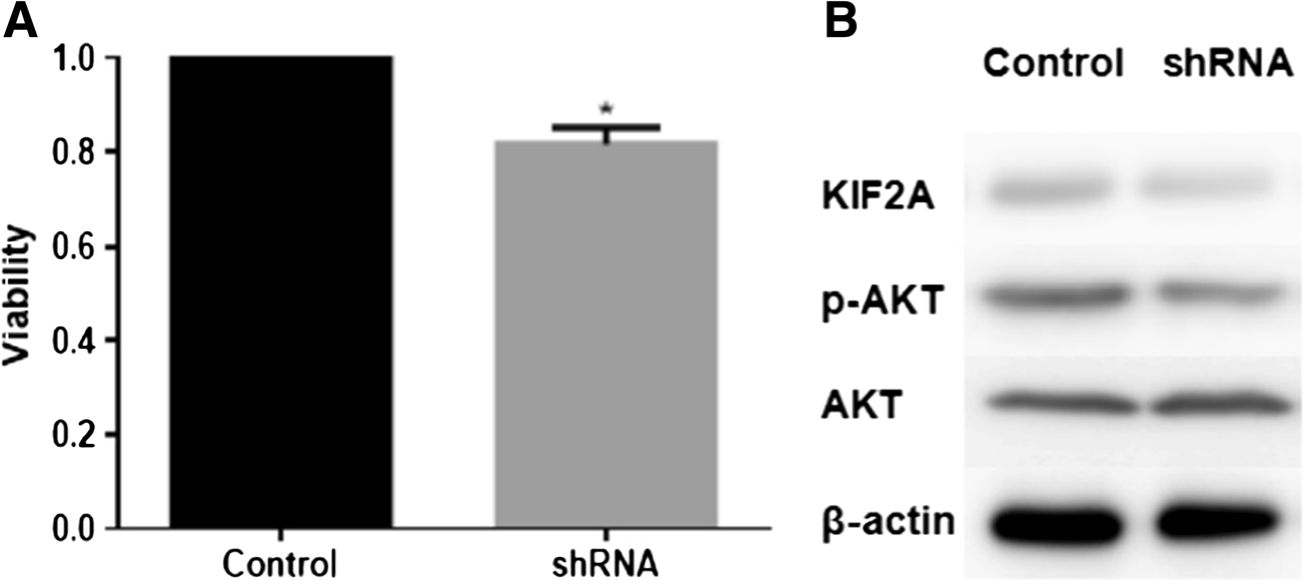
Downregulation of KIF2A inhibited lymphoma cell proliferation and was related to the AKT signaling pathway. **a** Silencing KIF2A by shRNA decreased the viability of SU-DHL-8 cells (*P* = 0.001). **b** Western blot showed that inhibition of KIF2A in SU-DHL-8 cell line was associated with a reduction in pAKT level

Using MTS assay, we determined the effect of shRNA-mediated downregulation of KIF2A on SU-DHL-8 cells. Molecular knockdown of KIF2A caused moderate growth inhibition in lymphoma cells (*P* < 0.05). Furthermore, the expression of pAKT was moderately reduced in SU-DHL-8 cells transfected with shRNA plasmids, whereas total AKT levels remained unchanged (Fig. 3).

## Discussion

DLBCL is an aggressive group of lymphoid malignancies with biological and clinical heterogeneity, involving various genetic and molecular alterations. No single biomarker can accurately and reliably identify this subset of DLBCL patients. Therefore, additional studies on biomarkers are required. Our study aimed to explore the expression of KIF2A in DLBCL, its correlation with clinical parameters, and the prognostic role of KIF2A expression in DLBCL. To our knowledge, this is the first study demonstrating the oncogenic potential of KIF2A in DLBCL.

KIF2A is a kinesin-13 family member. It is a microtubule-based motor protein and functions as a microtubule depolymerase. It is involved in several cellular processes including cytokinesis and mitosis [8]. Previously, Ganem et al. demonstrated that knockdown of KIF2A leads to monopolar spindle formation and chromosome mis-segregation, contributing to cell cycle inhibition [7]. Monopolar spindles and inhibition of cell proliferation were observed in KIF2A-deficient Xenopus eggs [8].

Recent studies reported the correlation between KIF2A expression and malignant tumors, including breast cancer, SCCOT, colorectal cancer, and ovarian cancer. A recent study explored the function of KIF2A during breast cancer development and progression. The expression of KIF2A was found to be remarkably higher in breast cancer tissue samples compared with corresponding adjacent tissues. Furthermore, the downregulation of KIF2A resulted in significant reduction of both cell growth and tumor migration and invasion, suggesting that KIF2A overexpression by breast cancer cells may alter key behaviors of tumor cells and lead to aberrant proliferation and metastasis [10]. In our cellular transfection model of DLBCL, moderately decreased tumor cell viability was observed when KIF2A expression was silenced, suggesting that KIF2A is involved in stimulating lymphoma cell growth. Another study showed that overexpression of KIF2A promoted metastasis and invasion of SCCOT, indicating that KIF2A overexpression is unfavorable for predicting the prognosis of SCCOT [20]. Notably, both in vivo and in vitro, treatment with KIF2A RNAi remarkably enhanced the sensitivity of SCCOT cells to 5-FU [21], suggesting that targeting KIF2A can overcome drug resistance and improve the treatment of tumors. In human epithelial ovarian cancer, overexpression of KIF2A was found in tumor tissues, and KIF2A conferred increased invasive and metastatic potential to tumor cells [13]. The Ki67 index is a critical prognostic parameter in various human cancers [22, 23]. Interestingly, a strong correlation was found between Ki67 and KIF2A expression in laryngeal squamous cell carcinoma [24], indicating that high KIF2A expression is closely associated with tumor aggressiveness. Therefore, KIF2A is involved in tumor growth, metastasis, and invasion, and confers chemoresistance in various human tumors.

In the present study, immunohistochemistry was used to evaluate KIF2A expression in 134 DLBCL tissue samples and 57 reactive hyperplasia samples. The prevalence of elevated KIF2A expression was increased in lymphoma tissues compared with reactive hyperplasia, indicating that KIF2A may contribute to the malignant transformation of DLBCL. These observations are consistent with those from other tumors [11, 20, 21]. Additionally, the overexpression of KIF2A was related to Ann Arbor stage and high-risk IPI stratification in patients with DLBCL. The univariate analyses showed that age, Ann Arbor stage, serum LDH level, high KIF2A expression, and IPI score were related to the survival time of patients with DLBCL. High KIF2A expression, serum LDH level, and IPI score maintained their prognostic value in multivariate analyses, indicating that dysregulation of KIF2A may be related to tumor progression in DLBCL. Therefore, our data suggest that KIF2A has oncogenic potential and may represent an independent prognostic marker for DLBCL.

Phosphatidylinositol 3-kinase (PI3K) is a member of the lipid kinase family. Aberrantly stimulated PI3K sequentially phosphorylates and activates protein kinase B (AKT) and downstream signaling cascades. These activated molecules regulate a wide range of crucial cellular processes, including survival, proliferation, apoptosis, invasion, and migration, conferring a survival advantage to various tumors [25, 26]. Increased PI3K/AKT signaling is common in various human tumors including DLBCL [27–29]. Interestingly, silencing KIF2A results in downregulation of PI3K/AKT mRNA and protein in SCCOT Tca8113 cells. This promotes apoptosis of these cells and indicates that PI3K/AKT is activated downstream of KIF2A [11]. Our findings were also consistent with a previous study which showed that blocking KIF2A induced a decrease in tumor cell viability, accompanied by downregulation of pAKT levels. Thus, these results suggest that KIF2A may contribute to tumorigenesis, at least partially by increasing PI3K/AKT signaling.

In conclusion, this study demonstrated the increased expression of KIF2A in DLBCL tissue. Increased KIF2A expression was considered as a negative biomarker of DLBCL. Targeting KIF2A expression may represent a novel therapeutic strategy for the treatment of DLBCL. However, additional studies in vitro and in vivo are necessary to clarify the underlying mechanism of KIF2A in DLBCL tumorigenesis.
